# Cauda equina syndrome following an uneventful spinal anaesthesia

**DOI:** 10.4103/0019-5049.60505

**Published:** 2010

**Authors:** Mamta Jain, Uma Srivastava, S Saxena, Anish K Singh, Aditya Kumar

**Affiliations:** Department of Anesthesiology and Critical Care, S. N. Medical College, Agra, India

Sir,

Serious neurological complications after spinal anaesthesia are rare but extremely distressing. We report a case of cauda equina syndrome following spinal anaesthesia in a patient who had no identifiable risk factor.

A young female patient was referred to our hospital with persistent weakness of lower limbs along with bladder and bowel incontinence following caesarean section done 16 days back under spinal anaesthesia. The records revealed that spinal anaesthesia was given in L2-3 interspace with 23 G single use Quincke needle in lateral position with 2.4 ml of 0.5% hyperbaric bupivacaine. Three attempts were made before successful spinal puncture. There was no paraesthesia or back pain during needle placement or drug injection, but blood stained CSF was aspirated at the first attempt. There was adequate surgical anaesthesia after 12 minutes and the 45 minutes of surgical duration was uneventful. In the postoperative period, the anaesthetic effect showed no improvement after 12 hours. She developed faecal and urinary incontinence the next day. A neuro-physician managed conservatively. Seeing no improvement even after 15 days, the relatives brought her to our hospital.

**Figure 1:**
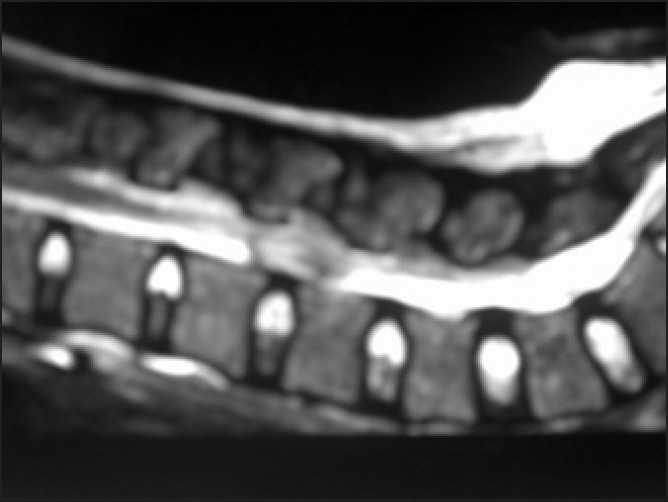
MRI of the spinal cord showing arachnoiditis

Examination by the neurosurgeon showed bilateral sensory motor deficit of both the limbs with impaired sensation to pinprick in the perineal region. A clinical diagnosis of cauda equina syndrome was made. Lumbosacral MRI showed thickening and clumping of cauda equina nerve roots at L2-3 level [Figure 1] along with post contrast (gadolinium) dural enhancement consistent with the diagnosis of arachnoiditis. There was no epidural abscess, haematoma or spinal canal stenosis. The patient was managed conservatively on heavy doses of steroids. Lower limb weakness gradually improved over three months (Grade 2 motor power).

Cauda equina syndrome is characterized by varying degree of saddle anaesthesia, sphincter dysfunction resulting in faecal incontinence, urinary retention and paraplegia. Although rare, case reports have shown an association with spinal anaesthesia.[1–3] Damage to nerve roots of cauda equina following spinal anaesthesia may occur due to compression, inflammation, stretching due to abnormal position, direct trauma, and spinal ischaemia or as a result of neurotoxicity of local anaesthetics.[2–4]

The most likely causes in our case could be neurotoxicity, haematoma or trauma to nerves or spinal cord. We presume that bleeding due to trauma could have resulted in haematoma formation and compression of nerve roots (this was not seen on MRI as it was done after about 18 days of spinal anaesthesia). The blood later would have been absorbed causing clumping of nerve roots and arachnoiditis.[4] Direct trauma to spinal cord or intraneural injection though can produce bilateral limb deficit were unlikely causes as the patient did not have any paraesthesia at the time of spinal anaesthesia.[5] Neurotoxicity of local anaesthetic is usually due to maldistribution[3] In the described patient, the thoracic sensory level block was bilateral and reached T8 level within 12 minutes, thus chances of neurotoxicity are minimal. Other possible causes of cauda equina syndrome after spinal anaesthesia were not identified in our patient.
